# Non‐Linear Changes in Face Availability During Naturalistic Playtime Across the First Years: Insights From Head‐Mounted Cameras and Automated Face Detection

**DOI:** 10.1111/desc.70121

**Published:** 2026-01-27

**Authors:** Teodor Y. Nikolov, Hana D'Souza

**Affiliations:** ^1^ Centre for Human Developmental Science, School of Psychology Cardiff University Cardiff UK

**Keywords:** egocentric vision, faces, head‐mounted cameras (headcams), infants, machine learning, toddlers

## Abstract

Faces provide crucial input for early development. This study leveraged innovations in wearable head‐mounted cameras (headcams; specifically, TinyExplorer gear) and automated face detection (RetinaFace) to characterise the everyday visual availability of faces during playtime in the home environment across the first years. Using a cross‐sectional developmental trajectory design, we collected egocentric headcam data from 29 young children across the first 3 years of life (2–30 months). The dataset comprised 1,891 minutes of video (over 5.5 million frames). We examined cross‐sectional developmental trajectories in face availability, spatial distribution, size and size variability. We observed distinct non‐linear changes in face availability across three vertically defined regions of the egocentric video (bottom/middle/top). In early infancy, faces were most common in the middle, with an initial steep decline followed by a modest increase after the first year. In the top region, during the second year, face presence increased and then decreased. The bottom region consistently showed low face presence. These findings suggest that the availability of faces is not only age‐dependent but also region‐specific, reflecting dynamic reorganisation of everyday visual input. Additionally, face size variability was greater in younger infants, consistent with caregiver‐driven interactions. We interpret our findings in the context of emerging motor abilities. By focusing on a specific activity (playtime), this study demonstrates how nuanced patterns can be detected using shorter recordings than in previous studies—enabling scalability and inclusivity of naturalistic research. These results offer new insights into early face availability and demonstrate the value of integrating naturalistic methods with automated analysis to advance developmental theory.

## Summary

- The availability of faces in egocentric views across the first 3 years shows non‐linear changes that vary by spatial region of the visual scene.
- Context‐constrained (playtime) egocentric recordings reveal subtle changes using relatively short recordings, facilitating more accessible head‐mounted camera data collection.
- Integration of head‐mounted cameras and automated face detection enables the scalable, spatially sensitive analysis of the real‐world visual availability of faces across the first years.

## 1 Background

Faces play a fundamental role in early development, shaping visual attention, social learning and caregiver–infant interactions. From birth, infants exhibit a preference for face‐like stimuli, which has been proposed to scaffold perceptual, cognitive and social development (Grossmann and Johnson 2007; Morton and Johnson 1991). Although there has been extensive research on face perception and social learning (for review, see Michel and Thiele 2025; Pascalis et al. 2011; Scott and Arcaro 2023), the egocentric visual experiences of faces in everyday environments have emerged as an important area of inquiry only more recently. The availability of lightweight head‐mounted cameras (headcams; Smith et al. 2015) in the past decade or so has provided novel opportunities to directly explore and characterise everyday face availability from the infant's own visual perspective.

To date, headcam studies of face availability typically fall into two categories, distinguished by their data collection context: home‐based and lab‐based. Most headcam studies are based in less structured, naturalistic home environments. Caregivers are typically asked to record 4–6 hours of video, capturing various everyday activities when infants are awake and alert. Using this design, cross‐sectional studies generally report a significant decline in the proportion of faces in infants’ visual scenes between 1 and 24 months (Fausey et al. 2016; Jayaraman et al. 2015). This decrease appears to be especially pronounced in the first 4 months of life (Jayaraman et al. 2013, 2015; Jayaraman et al. 2017; Jayaraman and Smith 2019; Sugden et al. 2013), a period during which infants’ visual scenes tend to contain many close‐up, upright faces (Jayaraman et al. 2015; Sugden and Moulson 2017). However, Long, Kachergis et al. (2022) captured a similar developmental pattern in a longitudinal study with even older infants (6–32 months, three infants tested weekly, SAYCam dataset; Sullivan et al. 2021). In contrast, a different pattern emerged in a cross‐sectional lab‐based study by Long, Sanchez et al. (2022), in which parents were instructed to play with pre‐selected objects as they typically would for 15–20 minutes. Albeit with a narrower age range (8, 12 and 16 months), this study did not replicate the developmental pattern of a decreasing presence of faces in visual scenes across age. If anything, a subtle U‐shaped trajectory for face availability was observed, with 12‐month‐olds encountering slightly fewer faces in their visual scenes compared to 8‐ and 16‐month‐olds.

In the current study, we aimed to reconcile these disparities by bridging the gap between less structured home studies and more structured laboratory investigations (for a discussion, see D'Souza and D'Souza 2024). To achieve this, we instructed caregivers to record headcam footage specifically during playtime (similarly to Long, Sanchez, et al. 2022) but situated within their natural home environments (similarly to Jayaraman et al. 2013, 2015, Jayaraman et al. 2017; Jayaraman and Smith 2019; Long, Kachergis, et al. 2022; Sugden et al. 2013). By strategically constraining certain contextual variables (in this case, type of activity) while preserving the ecological validity of home settings, our approach may provide an opportunity to detect subtler changes in the everyday visual availability of faces across the first years. Beyond mapping changes across age, we are interested in how these might coincide with the emergence of new motor abilities, such as transitions from lying to sitting and to independent locomotion, which may modulate the availability of faces in infants' visual scenes (Franchak et al. 2017, 2011; Karasik et al. 2013; Yamamoto et al. 2020).

**FIGURE 1:**
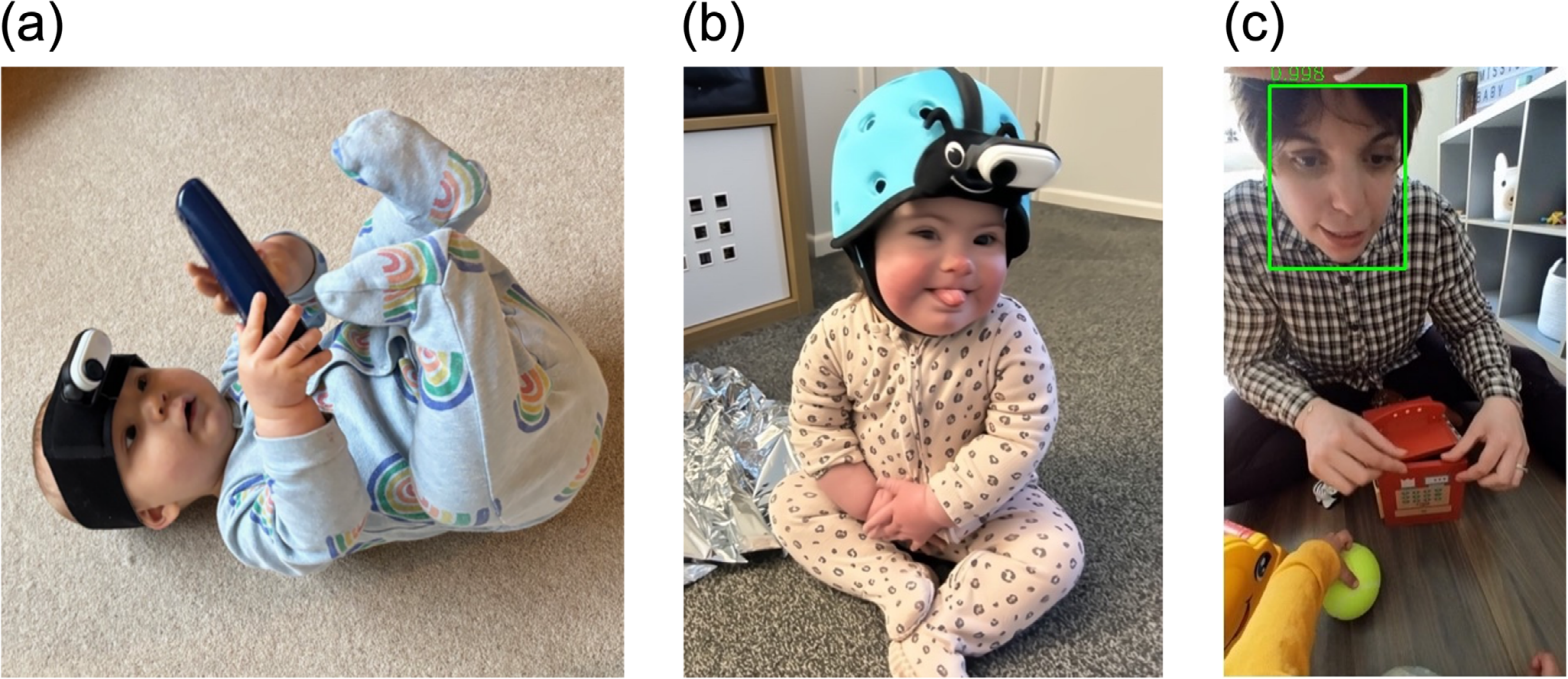
(a) TinyExplorer headband configuration suitable for younger infants; (b) TinyExplorer soft helmet configuration suitable for older infants and toddlers; (c) an example video frame with automated face detection output using RetinaFace; the number on the image shows the face detection confidence value.

**FIGURE 2:**
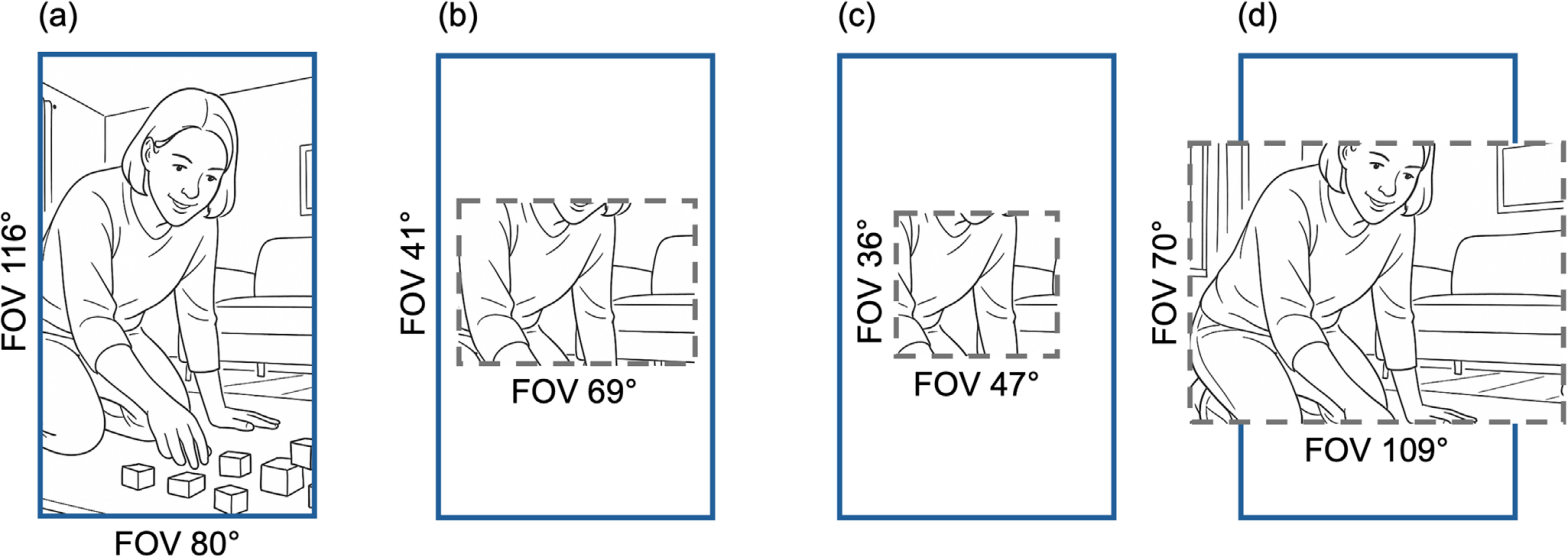
Field of view (FOV) comparisons across selected headcam studies: (a) current study (TinyExplorer gear; Nikolov et al. 2024, https://osf.io/95wvn/); (b) Fausey et al. (2016), Jayaraman et al. (2015), Jayaraman et al. (2017), Jayaraman and Smith (2019); (c) Long, Sanchez et al. (2022); (d) Long, Kachergis et al. (2022). Blue rectangles indicate the FOV of the current study, while grey dashed rectangles represent the FOV reported in each respective study. Illustrative scene is AI‐generated.

Building on recent advances in headcam technology (e.g., BabyView by Long et al. 2023; EgoActive by Geangu et al. 2023), we designed the TinyExplorer gear (Nikolov et al. 2024, https://osf.io/95wvn/; see Figure 1a,b) to fulfil the needs of the current study. This gear specifically prioritises lightweight modularity (allowing the headcam to be integrated with various hats and headbands) and ease of use, while maintaining high video and audio quality. Crucially, the TinyExplorer gear has a horizontal field of view (FOV) of 80° and a vertical FOV of 116°. Whilst this is comparable to the headcam studies reviewed above in the horizontal plane, it captures around three times as much FOV in the vertical plane (see Figure 2 for details). This enables us not only to replicate prior studies (by examining the middle region) but also to extend them in a key way: By examining how the distribution of faces across different regions of the footage (bottom, middle, top) shifts with age. Finally, to process the recordings, we employed the state‐of‐the‐art RetinaFace face‐detection algorithm (Deng et al. 2020). RetinaFace automatically detects faces in egocentric views of infants and young children with high accuracy (Nikolov et al. In press), while also seamlessly extracting additional valuable data: face size and position within each frame (see Figure 1c).

Taken together, by balancing ecological validity with methodological control, we leverage hardware and software innovations to characterise the availability of faces during contextually structured activity (playtime) within naturalistic home environments, integrating existing literature. Furthermore, exploring developmental patterns through the lens of emerging motor abilities offers further insights into the mechanisms driving developmental changes in infants’ visual scenes.

## 2 Methods

### 2.1 Participants

We analysed data from 29 infants and toddlers, aged 2–30 months (*M* = 16.0, SD = 8.4). Demographic information (following D'Souza et al. 2020; see Table 1) was collected from caregivers via a phone/online interview. Inclusion criteria were as follows: (1) being from monolingual English‐speaking households (≥95% English; 3 excluded); (2) no diagnosed neurodevelopmental condition; and (3) contributing at least 40 minutes of usable footage (two‐thirds of the 1‐hour target; two excluded).

**TABLE 1:** Participant characteristics.

Characteristic	Value
Sample size	29
Child age (months)	2–30; *M* = 16.0, SD = 8.4
Child sex	
Female	14
Male	15
Child ethnicity	
White	27
Black	0
Asian	0
Mixed	2
Other	0
Primary caregiver age (years)	26–45; *M* = 34.1, SD = 4.5
Secondary caregiver age (years)	25–54; *M* = 35.3, SD = 5.7 (one missing)
Children in household	1–3; *Mdn* = 2.0, *IQR* = 1.0
Adults in household	2–4; *Mdn* = 2.0, *IQR* = 0.0
Highest caregiver education	
Postgraduate degree	18
Undergraduate degree	9
Vocational/College	2
A‐levels	0
Secondary education	0
Highest caregiver occupation	
Managers, directors, senior officials	9
Professional occupations	8
Associate professionals/Technical	8
Administrative/Secretarial	1
Skilled trades	2
Caring/Leisure/Service	0
Sales/Customer service	0
Process/Plant/Machine operatives	1
Elementary occupations	0
Household income (£)	32,000–160,000; *Mdn* = 71,000, *IQR* = 34,000 (two missing)

Participants were approached through existing databases and opportunity sampling, including via social media, leaflets in local nurseries, events and word‐of‐mouth. Families were recruited with the goal of including one child at each monthly age point. We aimed to test each child within ±7 days of their monthly age mark to ensure balanced sampling across the early developmental period. In four cases, due to family circumstances, participants were tested outside the target window but were successfully replaced. Two additional participants started recording slightly outside the window (+1 day and +2 days) and despite multiple attempts, could not be replaced before the study end date; these children were retained in the final sample, and their inclusion did not alter the results.

Families were given a small gift (e.g., a T‐shirt, a book) and a £10 multi‐retailer gift voucher in return for their participation. In addition, participants with initially insufficient recording duration (*n* = 8) were invited to provide an extra recording and received an additional £5 voucher. Ethical approval was obtained from Cardiff University School of Psychology Ethics Committee (EC.23.08.08.6821GRA). Informed consent was obtained from caregivers.

### 2.2 Procedure

#### 2.2.1 TinyExplorer Gear

The TinyExplorer gear (Nikolov et al. 2024, https://osf.io/95wvn/; see Figure 1a,b), custom‐assembled headcam system, was used to record egocentric video data. The camera recorded at 50 frames per second (more individual frames within the same time period means that each frame captures a smaller portion of the movement, resulting in less blur). The analysed videos were vertically oriented (1080 × 1920 pixels; 9:16 aspect ratio) with a horizontal FOV of 80° and a vertical FOV of 116° (see Figure 1c).

#### 2.2.2 Video Recordings

Two ready‐to‐use TinyExplorer gears were posted to each family's home. Caregivers were instructed to put the TinyExplorer gear on their child's head and record until the camera runs out of battery (∼30 minutes per camera; ∼1 hour across two cameras). Instructions were provided on how to pause the camera if necessary. Families were asked to record at home on a typical day during playtime, when the child was ‘at their best’, and outside of meal and nap times. After the equipment was posted back, videos were exported and clipped to exclude segments with prolonged physical interference (e.g., touching or adjusting the camera or helmet for more than 5 seconds), significant misalignment of the camera view, or visible nudity beyond what would typically be seen at a public beach. Brief or minor physical interference that did not affect footage centrality were retained. Clipped sections accounted for a median of 4% of the total video duration per participant (*IQR* = 22.5%, range = 0%–80%). This resulted in a total of 5,673,918 usable frames across all participants (*Mdn* = 179,474; *IQR* = 43,939, range = 120,225–339,648). Finally, for all analyses, each frame was segmented into three parts (bottom, middle, top) to enable alignment of FOV with existing studies (see Figure 2).

#### 2.2.3 Motor Milestones

Within seven days of receiving the headcam equipment, caregivers completed a phone/online interview. This included questions about key motor milestones: sitting without support, standing with assistance, crawling on hands and knees, walking with assistance, standing alone and walking alone (WHO Multicentre Growth Reference Study Group 2006). Caregivers retrospectively reported the age in months at which their child first demonstrated these motor milestones. We calculated experience with each motor ability by subtracting the age at which they achieved the milestone from the child's current age.

### 2.3 Automated Annotation of Faces in Child's View

An automated face detection algorithm, RetinaFace (Deng et al. 2020), was applied to all frames to detect faces, outputting for each detection four coordinates defining the rectangular face region. A confidence threshold of 0.9 was used, meaning the model must be at least 90% certain in its prediction before labelling a region as a face. The algorithm and threshold choice were informed by our validation study (Nikolov et al. In press). In this validation study, we manually annotated a subsample of the same naturalistic playtime headcam data from 10 participants (age range 18–29 months; 34,013 frames), six of whom were also included in the current study's sample. The validation achieved 97% precision and 78% recall, with a tendency to slightly underestimate the proportion of faces relative to manual annotation. In the current study, applying this validated pipeline to the full dataset resulted in 19.63% of frames containing at least one face.

## 3 Analytical Approach

To investigate whether face availability, face size and face size variability changed with age and frame area, we divided each frame into three equally sized areas (bottom, middle, top). Faces were assigned to an area based on the vertical position of the centre of the bounding box. First, we evaluated whether recording duration was related to any of the variables of interest listed above, overall and for each frame area. Spearman's rank correlations indicated that none of these relationships were significant (*r_s_* s < 0.36, all *p*s > 0.05). Therefore, duration was not included in the following analyses for parsimony. Second, we fit both linear and generalized additive models (GAMs) to compare a literature‐grounded linear trajectory (e.g., Fausey et al. 2016) with a more flexible, data‐driven smooth curve (e.g., Long, Kachergis, et al. 2022). For these analyses, we used the *mgcv* package in R (Wood 2023). Linear models tested straightforward developmental hypotheses, whereas GAMs captured potential non‐linearities. Smooth terms were modelled using thin‐plate regression splines (Wood 2017, 2023). We began by fitting a linear model with age (in months), frame area (bottom, middle, top) and their interaction. We then fitted a second linear model, without the interaction term, to assess whether model fit changed. Following the same approach, we used smooth GAMs to explore potential non‐linear effects. We first fitted a smooth GAM with interaction, allowing separate smooth functions of age for each frame area, and then fitted a second model with a single smooth term for age. The basis dimension was set to *k* = 10 for each smooth. Basis dimension diagnostics (Wood 2017) revealed low *k*‐indices and significant *p* values; however, estimated degrees of freedom (*edf*) remained well below the basis limit (*k′* = 9), with a highest value of 5.22, indicating no evidence of overfitting. Therefore, *k* = 10 was retained for interpretability and parsimony. All models were first fitted using restricted maximum likelihood (REML) to report model fit statistics (adjusted *R* ^2^, deviance explained, AIC). For model comparisons, we refitted models with maximum likelihood (ML). Comparisons were conducted sequentially: (1) linear models with versus without the interaction; (2) smooth models with versus without the interaction; and finally, (3) the best‐fitting linear model compared with the best‐fitting smooth model. We compared nested models using likelihood ratio *χ* ^2^ tests for parametric terms and *F*‐tests when models differed in smooth terms. Model fit was further evaluated using deviance explained, AIC and adjusted *R* ^2^ to identify the best‐fitting models. In the Results section, we report only the best‐fitting models to streamline the text, while Supporting Information (Sections SI1–SI3) provides full comparison details, including parametric coefficients, smooth terms and model diagnostics. Finally, we conducted Mann–Whitney *U* tests to explore differences between motor milestone–defined groups, with statistical details provided in Supporting Information (Section SI4).

## 4 Results

### 4.1 Presence of Faces in the Child's View Across Age and Frame Area

**FIGURE 3:**
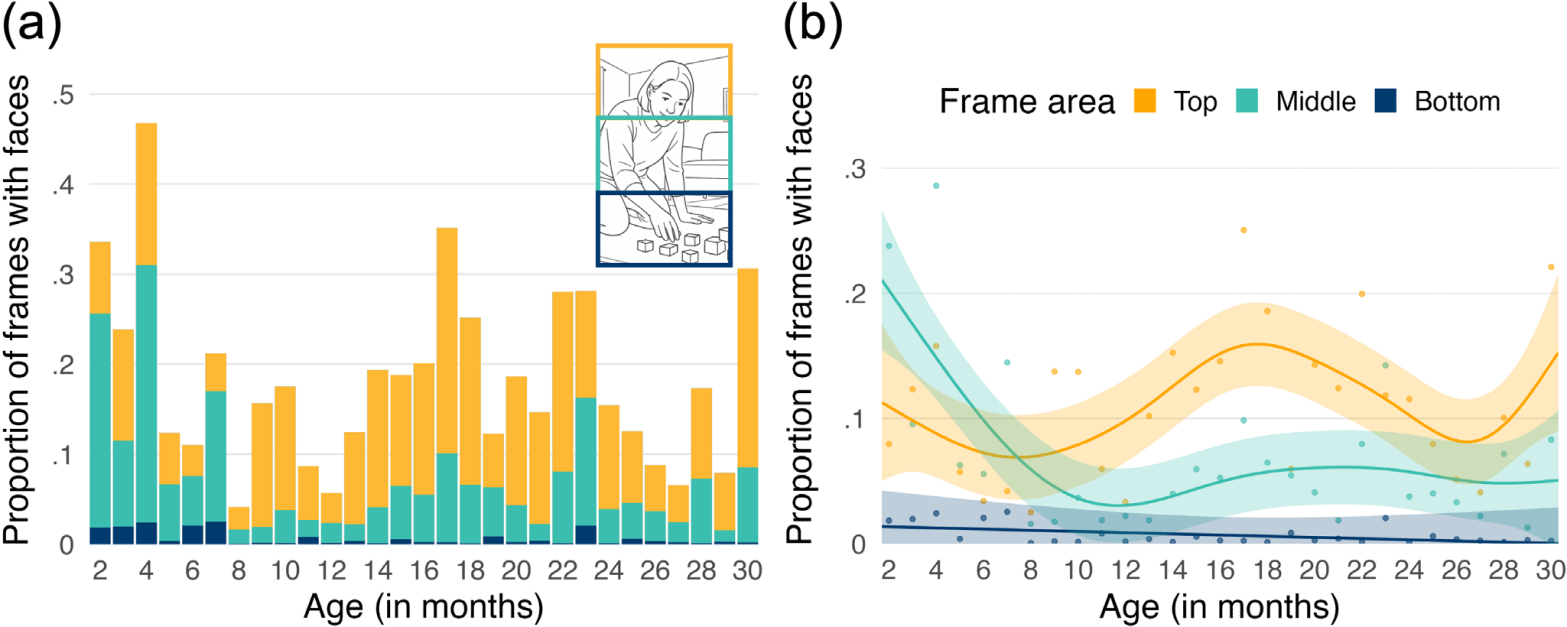
(a) Proportion of frames containing faces in each region of the visual frame (top, middle, bottom) across age (in months). Each bar represents a participant, with segments reflecting the relative contribution of each frame area to total face presence; (b) predicted proportion of frames containing faces across age (in months), plotted separately for each region of the visual frame (top, middle, bottom). Solid lines represent model predictions, shaded bands indicate 95% confidence intervals and points show observed values. Illustrative scene is AI‐generated.

The best‐fitted model explaining changes in the proportion of frames occupied by faces (see Figure 3a) was the smooth GAM with interaction (Figure 3b). This model explained 68.2% of the deviance, with an adjusted *R* ^2^ of 0.63, and an AIC of −295.7. In this model, the fixed effects for the frame area comparisons were significant (middle compared to bottom: *b* = 0.06, SE = 0.01, *t* = 5.81, *p* < 0.001; top compared to bottom: *b* = 0.10, SE = 0.01, *t* = 9.76, *p* < 0.001). As the bottom area was designated as the reference level in our models, we conducted pairwise comparisons using estimated marginal means (via the *emmeans* package; Lenth et al. 2025) to directly contrast the middle and top areas, revealing a significant difference*: b* = −0.10; SE = 0.02, *t* = −4.55, *p* < 0.001. The spline‐based smooth terms for age were statistically significant for the middle (*edf* = 4.05, *F* = 6.88, *p* < 0.001) and top (*edf* = 5.22, *F* = 2.84, *p* = 0.013) areas, but not for the bottom area (*edf* = 1.00, *F* = 0.28, *p* = 0.598) (see Figure 3b).

Taken together, as illustrated in Figure 3b, the results indicate differential changes in the three areas of the frames across age. Specifically, in the bottom area, there was no change in the proportion of faces across age. The middle area demonstrated a non‐linear change, characterised by an initial steep decrease in the proportion of frames with faces, followed by a slight increase in the presence of faces in this region after the first years of life. In the top area, most change was concentrated in the second year of life, showing an increase followed by a decrease.

### 4.2 Size of Faces in the Child's View Across Age

**FIGURE 4:**
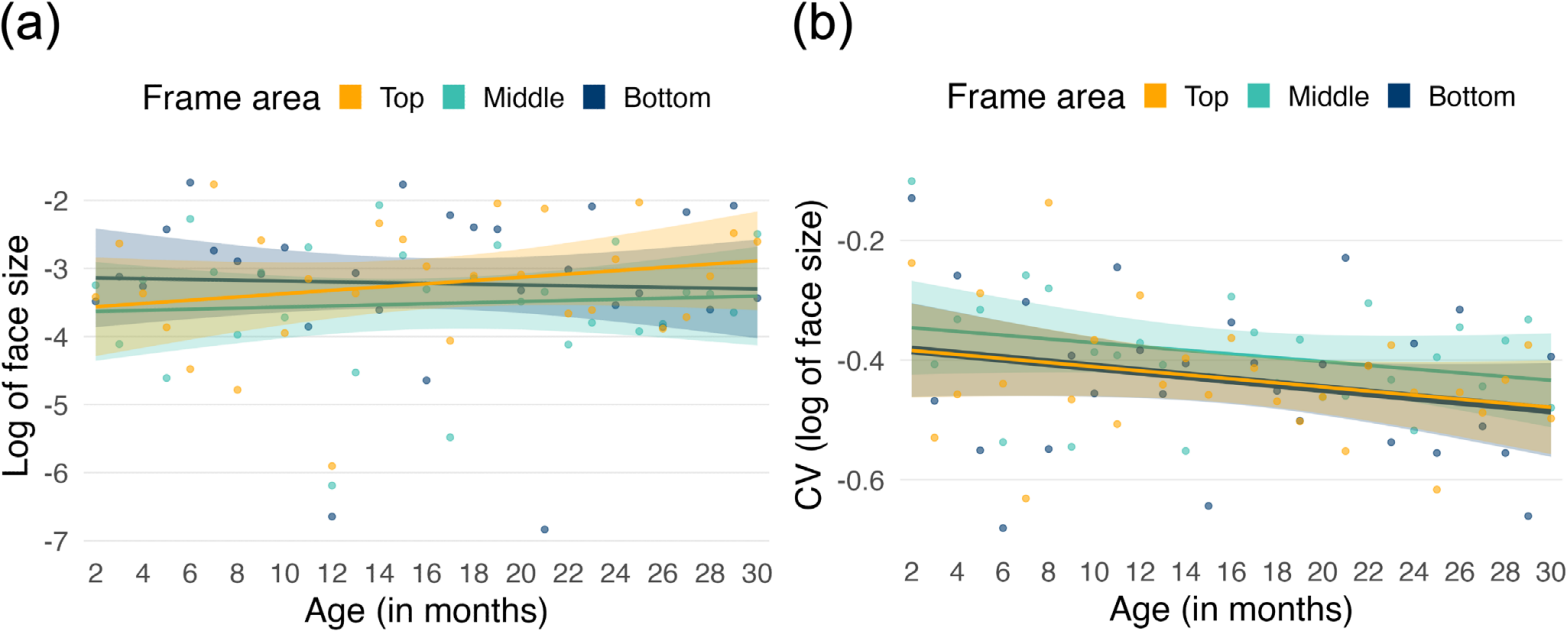
(a) Median face size (log‐transformed) across the top, middle and bottom areas of the frame as a function of age (in months). Observed values are shown as points, and the model predictions from a linear model are depicted as coloured lines with shaded 95% confidence intervals; (b) coefficient of variation (CV) of the log‐transformed proportion of frames containing a face is plotted as a function of age (in months) for each area (top, middle, bottom). Observed CV values are shown as points, and the model predictions from a linear model are depicted as coloured lines with shaded 95% confidence intervals.

We employed GAMs to examine whether the size of faces differs across age (Figure 4a). None of the effects in any of the models reached statistical significance (*p* > 0.05). In other words, the median size of faces did not differ across frame areas or age. Building on this, we next examined whether the variability of face size changes across age and area (Figure 4b). To carry out these analyses, for each frame, the face area (bounding box) was first normalised by the area of the frame region (1080 × 640 pixels) and then log‐transformed to improve the normality of the data distribution. The coefficient of variation (CV) was computed for each age (in months) and frame area (bottom, middle, top) as the standard deviation of the log‐transformed values divided by their mean. The linear model without the interaction showed the best fit (see Figure 4b). This model explained 9.54% of the deviance, with an adjusted *R* ^2^ of 0.063, and an AIC of −132.81. There was a significant effect of age (*b* < 0.01, SE < 0.01, *t* = −2.41, *p* = 0.018). The differences between the bottom and middle (*b* = 0.04, SE = 0.03, *t* = 1.51, *p* = 0.134) and bottom and top (*b* < 0.01, SE = 0.03, *t* = 0.06, *p* = 0.949) areas were not significant. As the bottom area was designated as the reference level in our models, we conducted pairwise comparisons using estimated marginal means (via the *emmeans* package) to directly contrast the middle and top areas—this was not significant (*b* = 0.04, SE = 0.03; *t* = 1.45, *p* = 0.151). Overall, these findings suggest that variability in the sizes of faces in view decreases significantly with age, regardless of area, and that this change is best captured by a linear function (see Figure 4b).

### 4.3 Visualising Motor Milestones

**FIGURE 5:**
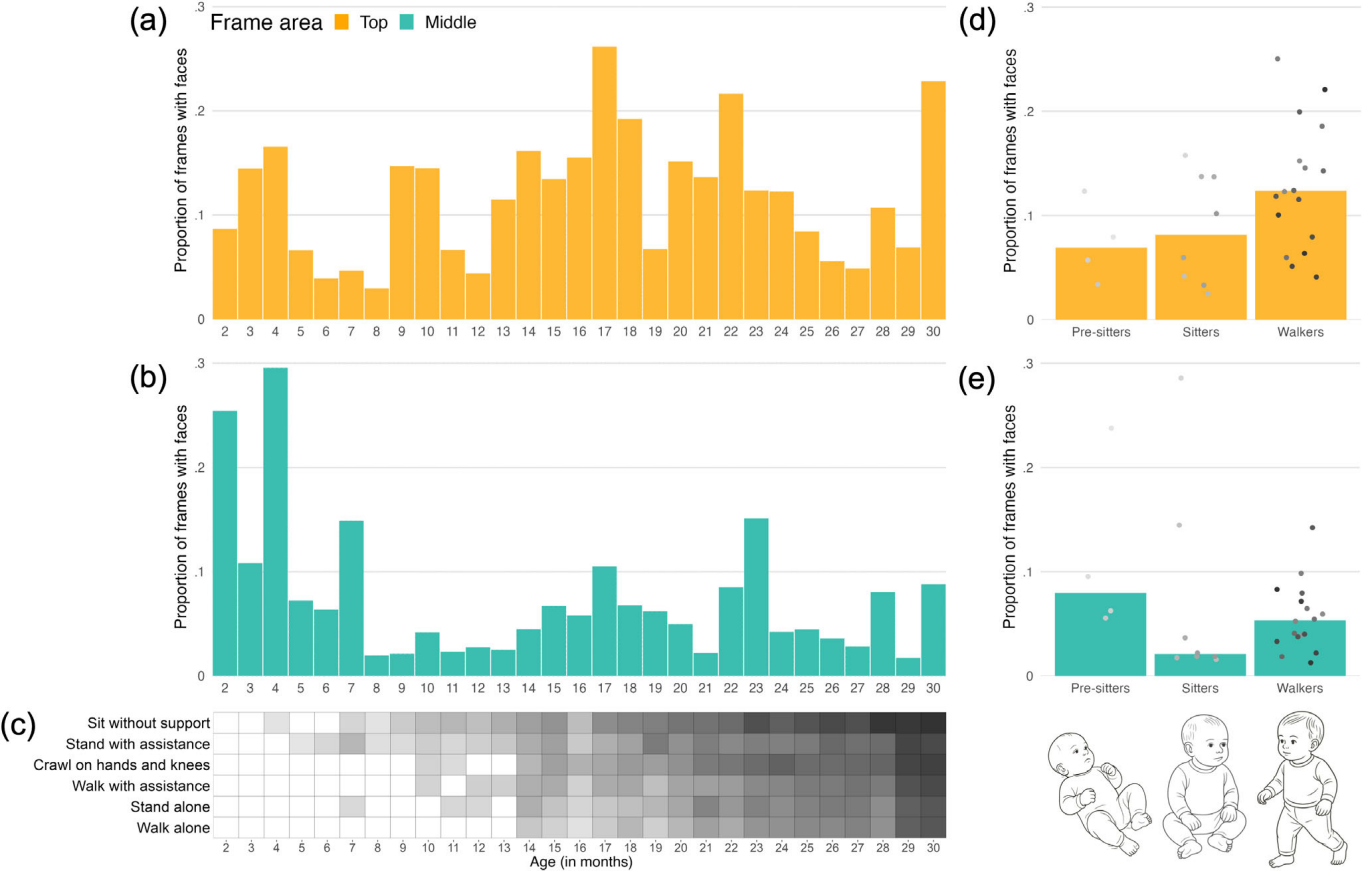
(a) Proportion of frames with faces across age in the top frame area, each bar corresponds to a participant; (b) proportion of frames with faces across age in the middle frame area, each bar corresponds to a participant; (c) motor milestones, each rectangle corresponds to one motor milestone for a particular participant. White indicates the ability has not yet emerged, grey indicates it has. Shading reflects experience with the ability (lighter to darker = less to more experience), calculated as the difference between the child's current age and the age at which they achieved the milestone. For example, the 8‐month‐old infant in this study had only recently begun sitting without support and standing with assistance, so their boxes are lighter, whereas the 29‐month‐old had much more motor experience, reflected in darker boxes; (d) data from (a) organised by motor ability group (pre‐sitters, sitters, walkers); (e) data from (b) organized by motor ability group (pre‐sitters, sitters, walkers). Pre‐sitters = unable to sit or walk; sitters = able to sit but unable to walk; walkers = able to sit and walk. Illustrative infant images are AI‐generated.

The non‐linear changes in the proportion of faces in the middle and top sections of frames identified above (Figure 3) suggest multiple points of re‐organisation during the first years of life. In this section, we explore the possibility that these may be associated with emerging motor abilities. In Figure 5, we provide visualisations aligning motor milestones with the proportion of faces in the top and middle sections of frames (as areas in which changes were detected; see above). Early on, there appears to be a notable decrease in the proportion of faces in the middle section of frames (Figure 5b), broadly corresponding to gaining expertise with independent sitting (Figure 5c). This period is followed by a gradual increase, particularly in the top area, observed at the onset of independent walking (Figure 5a–c). Additionally, Figure 5d,e visualises data categorised into pre‐sitters, independent sitters and independent walkers. Although preliminary non‐parametric Mann–Whitney *U* tests were not significant (Supporting Information, Section SI4), this analysis remains purely exploratory due to the extremely small group sizes. However, we hope these visualisations may inspire future hypothesis‐driven studies.

## 5 Discussion

The current study extends previous research by integrating recent innovations in wearable headcam technology (TinyExplorer gear; Nikolov et al. 2024, https://osf.io/95wvn/) and advanced machine‐learning algorithms (RetinaFace; Deng et al. 2020) to examine the everyday availability of faces in a playtime context in the first years of life, with the aim to integrate the existing literature. This approach revealed nuanced changes in the availability of faces across age, particularly highlighting distinct developmental trajectories across frame areas within the child's visual scenes, as well as decreases in the variability of face sizes. These results refine our current understanding of the early visual availability of faces (Fausey et al. 2016; Jayaraman et al. 2015; Long, Kachergis, et al. 2022; Long, Sanchez, et al. 2022), and align with the view that constraining the type of activity within the home environment may enable us to observe subtler developmental patterns.

Using a headcam with a wider vertical FOV than is typical in prior studies (see Figure 2) enabled us to carry out an analysis of the spatial distribution of faces across the top, middle and bottom sections of the frame. As the middle section is the most directly comparable to regions examined in earlier work, it allowed us to integrate our work with existing findings, whilst also examining the availability of faces in the upper or lower parts of the frame. We detected distinct non‐linear changes across age in the three frame areas (bottom, middle and top). The plotted patterns indicate that in young infants, faces were most often positioned in the middle area, with a steep decline across the first year of life. This was followed by a slight increase in the presence of faces in this region in the second year of life. In the top area, a similar but more pronounced increase emerged during the first half of the second year of life, followed by a decline. In contrast, the bottom area consistently showed a low proportion of faces, which is expected given its proximity to the child's body. It represents an area where faces are unlikely to appear. Taken together, these findings suggest that developmental changes in face availability are area‐specific, with dynamic, non‐linear age effects in the middle and top areas of the visual scene.

Our finding that in very young infants the proportion of faces was relatively high, with most faces being positioned in the middle, aligns with previous findings (e.g., Jayaraman et al. 2015). Specifically, it supports the interpretation that play at this age is largely face‐to‐face, with caregivers positioning their faces centrally in the infant's view, thereby shaping the visual content of infants’ views (Smith et al. 2018). This is also in line with our other finding that the sizes of faces in the views of young infants are more variable, consistent with interpretations that it is the caregivers who bring their faces in and out of view whilst young infants remain relatively static. Furthermore, the U‐shaped developmental trajectories in this study resemble a developmental trend observed in Long, Sanchez et al. (2022), where 12‐month‐olds were exposed to slightly fewer faces (albeit not significantly so) compared to 8‐ and 16‐month‐olds during lab‐based play sessions. Our results also broadly align with findings from Long, Kachergis et al. (2022), who investigated densely sampled recordings from three children from 6 to 32 months (SAYCam; Sullivan et al. 2021). They found that although a linear decrease in face availability was significant, quadratic terms relating children's age to the proportion of faces would provide a better fit to the data than linear terms alone. These results suggest that face availability changes with age but not necessarily in a linear fashion, particularly when playtime activity is considered.

Overall, the patterns in the top and middle areas suggest that face availability during playtime is not a fixed or monotonic process but instead follows a non‐linear trajectory across age. Periods of lower availability may represent transitional phases in which other elements of the environment occupy a greater proportion of visual scenes. Such variability underscores the importance of treating children's everyday input as dynamic and highlights the need to understand what processes contribute to these changes. The current observations align with theoretical accounts that emphasise the bidirectional relationship between visual experiences and emerging motor abilities (Campos et al. 2000; Gibson 1988; Smith et al. 2018). As infants learn to sit independently, their visual experiences might become less dominated by faces appearing in the middle and increasingly shaped by manual engagement with the surrounding environment that independent sitting enables (Mlincek et al. 2022). The increase in face presence in the top area after the first year of life may capture caregiver presence above the child whilst developing walking expertise (Franchak et al. 2017; Kretch et al. 2014), whereas the subsequent decline later in the second year of life may signal a shift towards greater autonomy. Although we did not observe statistically significant differences between (very small) motor milestone groups in the current study, we hope that the visualisations we provided will motivate further interest in how specific motor milestones may contribute to shifts in visual input (Adolph and Tamis‐LeMonda 2014; Karasik et al. 2012).

Notably, the fact that we observe non‐linear developmental patterns with comparatively shorter video recordings suggests that systematically constraining the type of activity in home environments may enable researchers to capture more nuanced changes. In the current study, caregivers were instructed to record during playtime when their child was ‘at their best’, and outside of meal and nap times, but face‐to‐face interaction was not emphasized. Importantly, the overall range of face availability we observed in the middle frame area is consistent with previous reports (e.g., Fausey et al. 2016), indicating that shorter playtime recordings are not systematically biased towards either including or excluding faces. This is also in line with our finding of no correlation between recording duration and the proportion of frames containing faces. However, it currently remains unclear how different activities precisely shape face availability. Thus, mapping the repertoire of everyday activities (de Barbaro 2019; Soderstrom and Wittebolle 2013; Tamis‐LeMonda et al. 2018) and examining the availability of faces in visual scenes as activity‐specific holds promise as a future research direction.

The possibility of meaningful, shorter recordings of selected activities also helps address one of the main barriers to participation in headcam studies: The significant number of hours families are typically asked to contribute. Reducing the required recording time makes participation more feasible for a wider range of families, ultimately increasing the accessibility and inclusivity of the research design. Shorter sessions are particularly beneficial for families with time constraints, neurodivergent children or those who may be hesitant to engage in long‐term studies. Our headcam design (TinyExplorer gear; Nikolov et al. 2024, https://osf.io/95wvn/) was specifically developed with these considerations in mind, with the future goal of expanding the participant pool by creating a user experience that is as seamless and unobtrusive as possible. This approach not only improves data collection logistics but also aligns with the broader goals of equity and inclusion in developmental research. It also represents a promising avenue for integrating naturalistic data into large‐scale cohort studies and clinical trials—domains where ecological validity is increasingly valued (Liu and Panagiotakos 2022). By capturing infants’ visual experiences in a minimally intrusive and standardised way, this design enables the examination of developmental processes in contextually rich environments.

There are some limitations of the current study that warrant discussion. First, while the automated detection algorithms preserve individual differences well (Nikolov et al. In press), they tend to systematically underestimate the presence of faces, especially in cases involving partial occlusion, unconventional angles or low‐light conditions. This makes the current approach more suitable for research questions focused on general developmental trends or broad measures of face availability over time, rather than fine‐grained analyses. Second, the present study utilised a cross‐sectional developmental approach, capturing snapshots of children's visual environments at different ages. Whilst this design allowed us to map broad developmental trajectories in face availability across the first years of life, highlighting non‐linear patterns across the developmental period, it needs to be followed by longitudinal work to trace individual trajectories. Finally, it is important to acknowledge the exploratory nature of the interpretations related to motor abilities. While we suggest a potential link between changes in the availability of faces in infants’ visual scenes and emerging motor abilities, these patterns should be interpreted with caution and in the context of a large body of literature demonstrating how posture and locomotion alter experiences (e.g., Franchak et al. 2017; Karasik et al. 2011, Karasik et al. 2013; Kretch et al. 2014; Long, Sanchez, et al. 2022; Luo and Franchak 2020; Mlincek et al. 2022; Thurman and Corbetta 2019; Yamamoto et al. 2020). Future research with larger samples recruited around the emergence of key motor milestones is needed to validate our preliminary observations and to better understand the mechanisms underlying these non‐linear developmental shifts, such as how changes in posture, manual exploration, locomotion and caregiver behaviour interact to shape visual scenes.

In summary, by integrating innovations in wearable headcam technology and advanced machine‐learning algorithms, this study characterises non‐linear changes in the availability of faces in infants’ views during a contextually constrained activity (playtime) in naturalistic home environments. These findings contribute to the foundation for future hypothesis‐driven research and may inform the design of intervention strategies that support early development.

## Funding

This work was supported by a James S. McDonnell Foundation (JSMF) Opportunity Award (https://doi.org/10.37717/2022‐3711) and a UKRI Future Leaders Fellowship (MR/X032922/1) awarded to H.D.

## Ethics Statement

All study procedures were approved by Cardiff University School of Psychology Ethics Committee (EC.23.08.08.6821GRA).

## Consent

Caregivers of all participants gave their informed consent prior to inclusion in the study.

## Conflicts of Interest

The authors declare no conflicts of interest.

## Supporting information




**Supporting File 1**: desc70121‐sup‐0001‐SupMat.docx

## Data Availability

The anonymised face detection output datasets generated and analysed during the current study are available from the corresponding authors on request.

## References

[desc70121-bib-0001] Adolph, K. E. , and C. S. Tamis‐LeMonda . 2014. “The Costs and Benefits of Development: The Transition From Crawling to Walking.” Child Development Perspectives 8, no. 4: 187–192. 10.1111/cdep.12085. 25774213 PMC4357016

[desc70121-bib-0002] Campos, J. J. , D. I. Anderson , M. A. Barbu‐Roth , E. M. Hubbard , M. J. Hertenstein , and D. Witherington . 2000. “Travel Broadens the Mind.” Infancy 1, no. 2: 149–219. 10.1207/S15327078IN0102_1. 32680291

[desc70121-bib-0003] de Barbaro, K. 2019. “Automated Sensing of Daily Activity: A New Lens Into Development.” Developmental Psychobiology 61, no. 3: 444–464. 10.1002/dev.21831. 30883745 PMC7343175

[desc70121-bib-0004] Deng, J. , J. Guo , E. Ververas , I. Kotsia , and S. Zafeiriou . 2020. “RetinaFace: Single‐Shot Multi‐Level Face Localisation in the Wild.” In 2020 IEEE/CVF Conference on Computer Vision and Pattern Recognition (CVPR) , 5202–5211. 10.1109/CVPR42600.2020.00525.

[desc70121-bib-0005] D'Souza, H. , and D. D'Souza . 2024. “Stop Trying to Carve Nature at Its Joints! The Importance of a Process‐Based Developmental Science for Understanding Neurodiversity.” Advances in Child Development and Behavior 66: 233–268. 10.1016/bs.acdb.2024.06.004. 39074923

[desc70121-bib-0006] D'Souza, H. , A. Lathan , A. Karmiloff‐Smith , and D. Mareschal . 2020. “Down Syndrome and Parental Depression: A Double Hit on Early Expressive Language Development.” Research in Developmental Disabilities 100: 103613. 10.1016/j.ridd.2020.103613. 32192950 PMC7167510

[desc70121-bib-0007] Fausey, C. M. , S. Jayaraman , and L. B. Smith . 2016. “From Faces to Hands: Changing Visual Input in the First Two Years.” Cognition 152: 101–107. 10.1016/j.cognition.2016.03.005. 27043744 PMC4856551

[desc70121-bib-0008] Franchak, J. M. , K. S. Kretch , and K. E. Adolph . 2017. “See and Be Seen: Infant–Caregiver Social Looking During Locomotor Free Play.” Developmental Science 21, no. 4: e12626. 10.1111/desc.12626. 29071760 PMC5920801

[desc70121-bib-0009] Franchak, J. M. , K. S. Kretch , K. C. Soska , and K. E. Adolph . 2011. “Head‐Mounted Eye Tracking: A New Method to Describe Infant Looking.” Child Development 82, no. 6: 1738–1750. 10.1111/j.1467-8624.2011.01670.x. 22023310 PMC3218200

[desc70121-bib-0010] Geangu, E. , W. A. P. Smith , H. T. Mason , et al. 2023. “EgoActive: Integrated Wireless Wearable Sensors for Capturing Infant Egocentric Auditory–Visual Statistics and Autonomic Nervous System Function ‘in the Wild’.” Sensors 23, no. 18: 7930. 10.3390/s23187930. 37765987 PMC10534696

[desc70121-bib-0011] Gibson, E. J. 1988. “Exploratory Behavior in the Development of Perceiving, Acting, and the Acquiring of Knowledge.” Annual Review of Psychology 39, no. 1: 1–42. 10.1146/annurev.ps.39.020188.000245.

[desc70121-bib-0012] Grossmann, T. , and M. H. Johnson . 2007. “The Development of the Social Brain in Human Infancy.” European Journal of Neuroscience 25, no. 4: 909–919. 10.1111/j.1460-9568.2007.05379.x. 17331189

[desc70121-bib-0013] Jayaraman, S. , C. M. Fausey , and L. B. Smith . 2013. “Visual Statistics of Infants' Ordered Experiences.” Journal of Vision 13, no. 9: 735. 10.1167/13.9.735.

[desc70121-bib-0014] Jayaraman, S. , C. M. Fausey , and L. B. Smith . 2015. “The Faces in Infant‐Perspective Scenes Change Over the First Year of Life.” PLoS ONE 10, no. 5: e0123780. 10.1371/journal.pone.0123780. 26016988 PMC4445910

[desc70121-bib-0015] Jayaraman, S. , C. M. Fausey , and L. B. Smith . 2017. “Why Are Faces Denser in the Visual Experiences of Younger Than Older Infants?” Developmental Psychology 53, no. 1: 38–49. 10.1037/dev0000230. 28026190 PMC5271576

[desc70121-bib-0016] Jayaraman, S. , and L. B. Smith . 2019. “Faces in Early Visual Environments Are Persistent Not Just Frequent.” Vision Research 157: 213–221. 10.1016/j.visres.2018.05.005. 29852210

[desc70121-bib-0017] Karasik, L. B. , K. E. Adolph , C. S. Tamis‐LeMonda , and A. L. Zuckerman . 2012. “Carry On: Spontaneous Object Carrying in 13‐Month‐Old Crawling and Walking Infants.” Developmental Psychology 48, no. 2: 389–397. 10.1037/a0026040. 22081880 PMC3580953

[desc70121-bib-0018] Karasik, L. B. , C. S. Tamis‐LeMonda , and K. E. Adolph . 2011. “Transition From Crawling to Walking and Infants' Actions With Objects and People.” Child Development 82, no. 4: 1199–1209. 10.1111/j.1467-8624.2011.01595.x. 21545581 PMC3163171

[desc70121-bib-0019] Karasik, L. B. , C. S. Tamis‐LeMonda , and K. E. Adolph . 2013. “Crawling and Walking Infants Elicit Different Verbal Responses From Mothers.” Developmental Science 17, no. 3: 388–395. 10.1111/desc.12129. 24314018 PMC3997624

[desc70121-bib-0020] Kretch, K. S. , J. M. Franchak , and K. E. Adolph . 2014. “Crawling and Walking Infants See the World Differently.” Child Development 85, no. 4: 1503–1518. 10.1111/cdev.12206. 24341362 PMC4059790

[desc70121-bib-0021] Lenth, R. V. , B. Banfai , B. Bolker , et al. 2025. “emmeans: Estimated Marginal Means, aka Least‐Squares Means (Version 1.11.0).” [Computer Software]. https://cran.r‐project.org/web/packages/emmeans/index.html.

[desc70121-bib-0022] Liu, F. , and D. Panagiotakos . 2022. “Real‐World Data: A Brief Review of the Methods, Applications, Challenges and Opportunities.” BMC Medical Research Methodology 22, no. 1: 287. 10.1186/s12874-022-01768-6. 36335315 PMC9636688

[desc70121-bib-0023] Long, B. , S. Goodin , G. Kachergis , et al. 2023. “The BabyView Camera: Designing a New Head‐Mounted Camera to Capture Children's Early Social and Visual Environments.” Behavior Research Methods 56, no. 4: 3523–3534. 10.3758/s13428-023-02206-1. 37656342 PMC12208306

[desc70121-bib-0024] Long, B. L. , G. Kachergis , K. Agrawal , and M. C. Frank . 2022. “A Longitudinal Analysis of the Social Information in Infants' Naturalistic Visual Experience Using Automated Detections.” Developmental Psychology 58, no. 12: 2211–2229. 10.1037/dev0001414. 36227287

[desc70121-bib-0025] Long, B. L. , A. Sanchez , A. M. Kraus , K. Agrawal , and M. C. Frank . 2022. “Automated Detections Reveal the Social Information in the Changing Infant View.” Child Development 93, no. 1: 101–116. 10.1111/cdev.13648. 34787894

[desc70121-bib-0026] Luo, C. , and J. M. Franchak . 2020. “Head and Body Structure Infants' Visual Experiences During Mobile, Naturalistic Play.” PLoS ONE 15, no. 11: e0242009. 10.1371/journal.pone.0242009. 33170881 PMC7654772

[desc70121-bib-0027] Michel, C. , and M. Thiele . 2025. “Revisiting the Object‐Processing Paradigm in the Study of Gaze Cues: What Two Decades of Research Have Taught Us About Infant Social Learning.” Infancy 30, no. 2: e70007. 10.1111/infa.70007. 39999280 PMC11856345

[desc70121-bib-0028] Mlincek, M. M. , E. J. Roemer , C. Kraemer , and J. M. Iverson . 2022. “Posture Matters: Object Manipulation During the Transition to Arms‐Free Sitting in Infants at Elevated vs. Typical Likelihood for Autism Spectrum Disorder.” Physical & Occupational Therapy in Pediatrics 42, no. 4: 351–365. 10.1080/01942638.2022.2027845. 35086427 PMC9203937

[desc70121-bib-0029] Morton, J. , and M. H. Johnson . 1991. “CONSPEC and CONLERN: A Two‐Process Theory of Infant Face Recognition.” Psychological Review 98, no. 2: 164–181. 10.1037/0033-295X.98.2.164. 2047512

[desc70121-bib-0031] Nikolov, T. Y. , C. Bocchetta , and H. D'Souza . 2024. “TinyExplorer Gear Build Manual.” [Manual]. https://osf.io/95wvn/.

[desc70121-bib-0032] Nikolov, T. Y. , J. Yurkovic‐Harding , T. Foldes , J. Bradshaw , Y.‐K. Lai , and H. D'Souza . In press. “Making Machine Learning Accessible for Developmental Science: The Case of Automated Face Detection.” Developmental Science . 10.1111/desc.70148 PMC13095670 42010051

[desc70121-bib-0033] Pascalis, O. , X. de Martin de Viviés , G. Anzures , et al. 2011. “Development of Face Processing.” WIREs Cognitive Science 2, no. 6: 666–675. 10.1002/wcs.146. 22039564 PMC3203018

[desc70121-bib-0034] Scott, L. S. , and M. J. Arcaro . 2023. “A Domain‐relevant Framework for the Development of Face Processing.” Nature Reviews Psychology 2, no. 3: 183–195. 10.1038/s44159-023-00152-5.

[desc70121-bib-0035] Smith, L. B. , S. Jayaraman , E. Clerkin , and C. Yu . 2018. “The Developing Infant Creates a Curriculum for Statistical Learning.” Trends in Cognitive Sciences 22, no. 4: 325–336. 10.1016/j.tics.2018.02.004. 29519675 PMC5866780

[desc70121-bib-0036] Smith, L. B. , C. Yu , H. Yoshida , and C. M. Fausey . 2015. “Contributions of Head‐Mounted Cameras to Studying the Visual Environments of Infants and Young Children.” Journal of Cognition and Development 16, no. 3: 407–419. 10.1080/15248372.2014.933430. 26257584 PMC4527180

[desc70121-bib-0037] Soderstrom, M. , and K. Wittebolle . 2013. “When Do Caregivers Talk? The Influences of Activity and Time of Day on Caregiver Speech and Child Vocalizations in Two Childcare Environments.” PLoS ONE 8, no. 11: e80646. 10.1371/journal.pone.0080646. 24260443 PMC3832484

[desc70121-bib-0038] Sugden, N. A. , M. I. Mohamed‐Ali , and M. C. Moulson . 2013. “I Spy With My Little Eye: Typical, Daily Exposure to Faces Documented From a First‐Person Infant Perspective.” Developmental Psychobiology 56, no. 2: 249–261. 10.1002/dev.21183. 24285109 PMC4262075

[desc70121-bib-0039] Sugden, N. A. , and M. C. Moulson . 2017. “Hey Baby, What's ‘Up’? One‐ and 3‐Month‐Olds Experience Faces Primarily Upright but Non‐Upright Faces Offer the Best Views.” Quarterly Journal of Experimental Psychology (2006) 70, no. 5: 959–969. 10.1080/17470218.2016.1154581. 26912287

[desc70121-bib-0040] Sullivan, J. , M. Mei , A. Perfors , E. Wojcik , and M. C. Frank . 2021. “SAYCam: A Large, Longitudinal Audiovisual Dataset Recorded From the Infant's Perspective.” Open Mind: Discoveries in Cognitive Science 5: 20–29. 10.1162/opmi_a_00039. 34485795 PMC8412186

[desc70121-bib-0041] Tamis‐LeMonda, C. S. , S. Custode , Y. Kuchirko , K. Escobar , and T. Lo . 2018. “Routine Language: Speech Directed to Infants During Home Activities.” Child Development 90, no. 6: 2135–2152. 10.1111/cdev.13089. 29766498

[desc70121-bib-0042] Thurman, S. L. , and D. Corbetta . 2019. “Changes in Posture and Interactive Behaviors as Infants Progress From Sitting to Walking: A Longitudinal Study.” Frontiers in Psychology 10: 822. 10.3389/fpsyg.2019.00822. 31031682 PMC6473077

[desc70121-bib-0030] WHO Multicentre Growth Reference Study Group . 2006. “WHO Motor Development Study: Windows of Achievement for Six Gross Motor Development Milestones.” Acta Paediatrica 95, no. S450: 86–95. 10.1111/j.1651-2227.2006.tb02379.x. 16817682

[desc70121-bib-0043] Wood, S. N. 2017. Generalized Additive Models: An Introduction With R. 2nd ed. Chapman and Hall/CRC.

[desc70121-bib-0044] Wood, S. N. 2023. “mgcv: Mixed GAM Computation Vehicle With Automatic Smoothness Estimation (Version 1.9‐1).” [Computer Software]. https://cran.r‐project.org/web/packages/mgcv/index.html.

[desc70121-bib-0045] Yamamoto, H. , A. Sato , and S. Itakura . 2020. “Transition From Crawling to Walking Changes Gaze Communication Space in Everyday Infant‐Parent Interaction.” Frontiers in Psychology 10: 2987. 10.3389/fpsyg.2019.02987. 32116864 PMC7025586

