# Supplementary material for: Non‐Linear Changes in Face Availability During Naturalistic Playtime Across the First Years: Insights From Head‐Mounted Cameras and Automated Face Detection
Source: Dev Sci. 2026 Jan 27;29(2):e70121. doi: 10.1111/desc.70121 (PMC12836453; doi:10.1111/desc.70121)
Supplement: Supplementary file 1 — Supporting File 1: desc70121‐sup‐0001‐SupMat.docx [file DESC-29-e70121-s001.docx]

**Supporting Information**

**SI1. Analyses of Face Availability (Proportion of Frames)**

**Table SI1.1**

*Model Fit Statistics for Generalized Additive Models Predicting Face Availability (Proportion of Frames)*

| Model | Formula | Adj. *R²* | Deviance explained | AIC |
| --- | --- | --- | --- | --- |
| Linear with interaction | Face Availability ~ Age × Frame Area | .44 | 47.2% | –270.01 |
| Linear without interaction | Face Availability ~ Age + Frame Area | .40 | 42.3% | –266.36 |
| **Smooth with interaction** | **Face Availability ~ *s*(Age, by = Frame Area, k = 10) + Frame Area** | **.63** | **68.2%** | **–295.7** |
| Smooth without interaction | Face Availability ~ *s*(Age, k = 10) + Frame Area | .47 | 50.4% | –271.68 |

*Note.* Age is measured in months. Frame Area refers to regions of the frame (bottom, middle, top). Face Availability denotes the proportion of frames in which a face was detected within each frame area. The best fitting model is shown in bold.

**Table SI1.2**

*ANOVA Model Comparisons for Generalized Additive Models Predicting Face Availability (Proportion of Frames)*

| Comparison | *F/χ²* | *df* | *p* |
| --- | --- | --- | --- |
| Linear with interaction vs. Linear without interaction | 0.02 | 2 | .024 |
| Smooth with interaction vs. Smooth without interaction | 4.83 | 8.60 | <.001 |
| Linear with interaction vs. Smooth with interaction | 4.33 | 10.9 | <.001 |

*Note.* Results are from likelihood ratio tests (LRTs) based on ML-fitted GAMs. Comparisons differing only in parametric terms were evaluated with *χ²* tests, whereas comparisons involving penalized smooth terms were evaluated with approximate *F*-tests.

**Table SI1.3**

*Parametric Terms for Generalized Additive Models Predicting Face Availability (Proportion of Frames)*

| Model | Term | Estimate (*b*) | *SE* | *t* | *p* |
| --- | --- | --- | --- | --- | --- |
| Linear with interaction | Intercept (bottom) | 0.01 | 0.02 | 0.73 | .467 |
|  | Age (bottom) | –0.00 | 0.00 | –0.43 | .668 |
|  | Area (middle vs. bottom) | 0.10 | 0.03 | 3.58 | <.001 |
|  | Area (top vs. bottom) | 0.07 | 0.03 | 2.66 | .009 |
|  | Age × Area (middle) | –0.00 | 0.00 | –1.57 | .121 |
|  | Age × Area (top) | 0.00 | 0.00 | 1.15 | .253 |
|  | Pairwise contrast (middle vs. top) | –0.04 | 0.01 | –3.21 | .002 |
| Linear without interaction | Intercept (bottom) | 0.02 | 0.01 | 1.27 | .208 |
|  | Age (bottom) | –0.00 | 0.00 | –1.05 | .296 |
|  | Area (middle vs. bottom) | 0.06 | 0.01 | 4.58 | <.001 |
|  | Area (top vs. bottom) | 0.10 | 0.01 | 7.69 | <.001 |
|  | Pairwise contrast (mid–top) | –0.04 | 0.01 | –3.11 | .003 |
| **Smooth with interaction** | Intercept (bottom) | 0.01 | 0.01 | 0.93 | .358 |
|  | Area (middle vs. bottom) | 0.06 | 0.01 | 5.81 | <.001 |
|  | Area (top vs. bottom) | 0.10 | 0.01 | 9.76 | <.001 |
|  | Pairwise contrast (middle vs. top) | –0.10 | 0.02 | –4.55 | <.001 |
| Smooth without interaction | Intercept (bottom) | 0.01 | 0.01 | 0.77 | .442 |
|  | Area (middle vs. bottom) | 0.06 | 0.01 | 4.85 | <.001 |
|  | Area (top vs. bottom) | 0.10 | 0.01 | 8.14 | <.001 |
|  | Pairwise contrast (middle vs. top) | –0.04 | 0.01 | –3.30 | .002 |

*Note.* Estimates (*b*), standard errors (SE), *t* values, and *p* values are from REML-fitted linear GAMs. Pairwise contrasts were estimated with emmeans (evaluated at the mean of age). The best fitting model is shown in bold.

**Table SI1.4**

*Smooth Terms and Basis Dimension Diagnostics for Generalized Additive Models Predicting Face Availability (Proportion of Frames)*

| Model | Smooth term | *edf* | Ref.df | *F* | *p* (smooth) | *k* | *k′* | *k*-index | *p* (k check) |
| --- | --- | --- | --- | --- | --- | --- | --- | --- | --- |
| Smooth with interaction | *s*(Age): bottom | 1.00 | 1.00 | 0.28 | .598 | 10 | 9 | 0.76 | .005 |
| Smooth with interaction | *s*(Age): middle | 4.05 | 5.00 | 6.88 | <.001 | 10 | 9 | 0.76 | .025 |
| Smooth with interaction | *s*(Age): top | 5.22 | 6.33 | 2.84 | .013 | 10 | 9 | 0.76 | .005 |
| Smooth without interaction | *s*(Age) | 3.98 | 4.91 | 2.31 | .061 | 10 | 9 | 0.98 | .400 |

*Note.* Smooth terms report estimated degrees of freedom (*edf*), reference degrees of freedom (Ref.df), F statistics, and approximate significance. k′, k-index, and p (k check) values are from gam.check diagnostics. We specified a basis dimension of k = 10. Although k-indices were < 1 with significant p values, increasing to k = 20 yielded nearly identical *edf* and smooth estimates, with no change in k-index. This suggests the diagnostic warning reflects the small sample size and multiple by-factor smooths rather than underspecified k. Consistent with recommendations (Wood, 2017), we retained k = 10 as results were stable across specifications.

**SI2. Analyses of Face Size (Log-Transformed Median Bounding Box Proportion)**

**Table SI2.1**

*Model Fit Statistics for Generalized Additive Models Predicting Face Size (Log-Transformed Median Bounding Box Proportion)*

| Model | Formula | Adj. *R²* | Deviance explained | AIC |
| --- | --- | --- | --- | --- |
| Linear with interaction | Face Size ~ Age × Frame Area | −.02 | 3.5% | 258.34 |
| **Linear without interaction** | **Face Size ~ Age + Frame Area** | **−.01** | **2.5%** | **255.27** |
| Smooth with interaction | Face Size ~ *s*(Age, by = Frame Area, k = 10) + Frame Area | −.02 | 3.5% | 258.34 |
| Smooth without interaction | Face Size ~ *s*(Age, k = 10) + Frame Area | −.01 | 2.5% | 255.27 |

*Note.* Adjusted *R²*, deviance explained, and AIC are reported from GAMs fitted using restricted maximum likelihood (REML). The best fitting model is shown in bold.

**Table SI2.2**

*Likelihood Ratio Test Comparisons for Generalized Additive Models Predicting Face Size (Log-Transformed Median Bounding Box Proportion)*

| Comparison | *F/χ²* | *df* | *p* |
| --- | --- | --- | --- |
| Linear with interaction vs. Linear without interaction | 0.91 | 2 | .648 |
| Smooth with interaction vs. Smooth without interaction | 0.43 | 2 | .649 |
| Linear without interaction vs. Smooth without interaction | 0.61 | 0 | <.001 |

*Note.* Results are based on likelihood ratio tests (LRTs) using maximum likelihood (ML)–fitted GAMs. Comparisons differing only in parametric terms were evaluated with *χ²* tests, whereas comparisons involving penalized smooth terms were evaluated with approximate *F*-tests.

**Table SI2.3**

*Parametric Terms for Generalized Additive Models Predicting Face Size (Log-Transformed Median Bounding Box Proportion)*

| Model | Term | Estimate (*b*) | *SE* | *t* | *p* |
| --- | --- | --- | --- | --- | --- |
| Linear with interaction | Intercept (bottom) | −3.13 | 0.41 | −7.64 | <.001 |
|  | Age (bottom) | −0.01 | 0.02 | −0.26 | .799 |
|  | Area (middle vs. bottom) | −0.52 | 0.58 | −0.90 | .371 |
|  | Area (top vs. bottom) | −0.48 | 0.58 | −0.84 | .406 |
|  | Age × Area (middle) | 0.01 | 0.03 | 0.43 | .666 |
|  | Age × Area (top) | 0.03 | 0.03 | 0.93 | .354 |
|  | Pairwise contrast (middle vs. top) | −0.29 | 0.27 | −1.09 | .278 |
| **Linear without interaction** | Intercept (bottom) | −3.36 | 0.28 | −11.98 | <.001 |
|  | Age (bottom) | 0.01 | 0.01 | 0.68 | .501 |
|  | Area (middle vs. bottom) | −0.30 | 0.27 | −1.12 | .266 |
|  | Area (top vs. bottom) | −0.01 | 0.27 | −0.02 | .984 |
|  | Pairwise contrast (middle vs. top) | −0.29 | 0.27 | −1.10 | .275 |
| Smooth with interaction | Intercept (bottom) | −3.22 | 0.19 | −16.98 | <.001 |
|  | Area (middle vs. bottom) | −0.30 | 0.27 | −1.11 | .269 |
|  | Area (top vs. bottom) | −0.01 | 0.27 | −0.02 | .984 |
|  | Pairwise contrast (middle vs. top) | −0.29 | 0.27 | −1.09 | .278 |
| Smooth without interaction | Intercept (bottom) | −3.22 | 0.19 | −17.10 | <.001 |
|  | Area (middle vs. bottom) | −0.30 | 0.27 | −1.12 | .266 |
|  | Area (top vs. bottom) | −0.01 | 0.27 | −0.02 | .984 |
|  | Pairwise contrast (middle vs. top) | −0.29 | 0.27 | −1.10 | .275 |

*Note.* Estimates (*b*), standard errors (SE), *t* values, and *p* values are from REML-fitted linear GAMs. Pairwise contrasts were estimated with emmeans (evaluated at the mean of age). The best fitting model is shown in bold.

**Table SI2.4**

*Smooth Term Estimates and Basis Dimension Diagnostics for Generalized Additive Models Predicting Face Size (Log-Transformed Median Bounding Box Proportion)*

| Model | Smooth term | *edf* | Ref.df | *F* | *p* (smooth) | *k* | *k′* | *k*-index | *p* (k check) |
| --- | --- | --- | --- | --- | --- | --- | --- | --- | --- |
| Smooth with interaction | *s*(Age): bottom | 1.00 | 1.00 | 0.07 | .799 | 10 | 9 | 0.80 | .035 |
| Smooth with interaction | *s*(Age): middle | 1.00 | 1.00 | 0.13 | .722 | 10 | 9 | 0.80 | .040 |
| Smooth with interaction | *s*(Age): top | 1.00 | 1.00 | 1.13 | .292 | 10 | 9 | 0.80 | .030 |
| Smooth without interaction | *s*(Age) | 1.00 | 1.00 | 0.46 | .502 | 10 | 9 | 0.81 | .035 |

*Note.* Smooth terms report estimated degrees of freedom (*edf*), reference degrees of freedom (Ref.df), F statistics, and approximate significance. k′, k-index, and p (k check) are from gam.check. We set the basis dimension to k = 10 for all smooths. Although the k-index values were < 1 with small p values, the *edf* remained well below the basis limit (k′) and refitting with k = 20 produced essentially identical *edf*, coefficients, and fitted values, with k-indices unchanged (≈ 0.80). This pattern indicates that the warnings do not reflect an underspecified k but rather limited information per by-factor smooth and modest sample size. Following recommendations (Wood, 2017), we retained k = 10 for parsimony and comparability across models.

**SI3. Analyses of Face Size Variability (Coefficient of Variation of Log-Transformed Bounding Box Proportion)**

**Table SI3.1**

*Model Fit Statistics for Generalized Additive Models Predicting Face Size Variability (Coefficient of Variation of Log-Transformed Bounding Box Proportion)*

| Model | Formula | Adj. *R²* | Deviance explained | AIC |
| --- | --- | --- | --- | --- |
| Linear with interaction | Face Size Variability ~ Age × Frame Area | .04 | 9.6% | −128.83 |
| **Linear without interaction** | **Face Size Variability ~ Age + Frame Area** | **.06** | **9.5%** | **−132.81** |
| Smooth with interaction | Face Size Variability ~ *s*(Age, by = Interest Area, k = 10) + Frame Area | .05 | 10.6% | −128.47 |
| Smooth without interaction | Face Size Variability ~ *s(*Age, k = 10) + Frame Area | .06 | 9.5% | −132.81 |

*Note.* Adjusted *R²*, deviance explained, and AIC are reported from GAMs fitted using restricted maximum likelihood (REML). The best fitting model is shown in bold.

**Table SI3.2**

*ANOVA Model Comparisons for Generalized Additive Models Predicting Face Size Variability (Coefficient of Variation of Log-Transformed Bounding Box Proportion)*

| Comparison | *F/χ²* | *df* | *p* |
| --- | --- | --- | --- |
| Linear with interaction vs. Linear without interaction | .00 | 2 | .992 |
| Smooth with interaction vs. Smooth without interaction | .01 | 2 | .992 |
| Linear without interaction vs. Smooth without interaction | .43 | 0 | <.001 |

*Note.* Results are based on likelihood ratio tests (LRTs) using maximum likelihood (ML)–fitted GAMs. Comparisons differing only in parametric terms were evaluated with *χ²* tests, whereas comparisons involving penalized smooth terms were evaluated with approximate *F*-tests.

**Table SI3.3**

*Parametric Terms for Generalized Additive Models Predicting Face Size Variability (Coefficient of Variation of Log-Transformed Bounding Box Proportion)*

| Model | Term | Estimate (*b*) | *SE* | *t* | *p* |
| --- | --- | --- | --- | --- | --- |
| Linear with interaction | Intercept (bottom) | −0.38 | 0.04 | −8.50 | <.001 |
|  | Age (bottom) | −0.00 | 0.00 | −1.46 | .147 |
|  | Area (middle vs. bottom) | 0.04 | 0.06 | 0.58 | .564 |
|  | Area (top vs. bottom) | −0.00 | 0.06 | −0.02 | .984 |
|  | Age × Area (middle) | 0.00 | 0.00 | 0.13 | .898 |
|  | Age × Area (top) | 0.00 | 0.00 | 0.06 | .955 |
|  | Pairwise contrast (middle vs. top) | 0.04 | 0.03 | 1.43 | .156 |
| **Linear without interaction** | Intercept (bottom) | −0.38 | 0.03 | −12.58 | <.001 |
|  | Age (bottom) | −0.00 | 0.00 | −2.41 | .018 |
|  | Area (middle vs. bottom) | 0.04 | 0.03 | 1.51 | .134 |
|  | Area (top vs. bottom) | 0.00 | 0.03 | 0.06 | .949 |
|  | Pairwise contrast (middle vs. top) | 0.04 | 0.03 | 1.45 | .151 |
| Smooth with interaction | Intercept (bottom) | −0.43 | 0.02 | −21.21 | <.001 |
|  | Area (middle vs. bottom) | 0.04 | 0.03 | 1.50 | .137 |
|  | Area (top vs. bottom) | 0.00 | 0.03 | 0.06 | .950 |
|  | Pairwise contrast (middle vs. top) | 0.03 | 0.03 | 1.00 | .321 |
| Smooth without interaction | Intercept (bottom) | −0.43 | 0.02 | −21.40 | <.001 |
|  | Area (middle vs. bottom) | 0.04 | 0.03 | 1.51 | .134 |
|  | Area (top vs. bottom) | 0.00 | 0.03 | 0.06 | .949 |
|  | Pairwise contrast (middle vs. top) | 0.04 | 0.03 | 1.45 | .151 |

*Note.* Estimates (*b*), standard errors (SE), *t* values, and *p* values are from REML-fitted linear GAMs. Pairwise contrasts were estimated with emmeans (evaluated at the mean of age). The best fitting model is shown in bold.

**Table SI3.4**

*Smooth Term Estimates and Basis Dimension Diagnostics for Generalized Additive Models Predicting Face Size Variability (Coefficient of Variation of Log-Transformed Bounding Box Proportion)*

| Model | Smooth term | *edf* | Ref.df | *F* | *p* (smooth) | *k* | *k′* | *k*-index | *p* (k check) |
| --- | --- | --- | --- | --- | --- | --- | --- | --- | --- |
| Smooth with interaction | *s*(Age): bottom | 1.00 | 1.00 | 2.16 | .146 | 10 | 9 | 0.84 | .065 |
| Smooth with interaction | *s*(Age): middle | 1.39 | 1.68 | 0.83 | .318 | 10 | 9 | 0.84 | .065 |
| Smooth with interaction | *s*(Age): top | 1.00 | 1.00 | 1.93 | .169 | 10 | 9 | 0.84 | .055 |
| Smooth without interaction | *s*(Age) | 1.00 | 1.00 | 5.82 | .018 | 10 | 9 | 0.85 | .050 |

*Note.* Smooth terms report estimated degrees of freedom (*edf*), reference degrees of freedom (Ref.df), F statistics, and approximate significance. k′, k-index, and p (k check) are from gam.check. We set the basis dimension to k = 10 for all smooths. Although the k-index values were < 1 with small p values, the *edf* remained well below the basis limit (k′) and refitting with k = 20 produced essentially identical *edf*, coefficients, and fitted values, with k-indices unchanged (≈ 0.84–0.85). This pattern indicates that the warnings do not reflect an underspecified k but rather limited information per by-factor smooth and modest sample size. Following recommendations (Wood, 2017), we retained k = 10 for parsimony and comparability across models.

**SI4. Analyses of Motor Milestones**

**Table SI4**

*Mann–Whitney U Tests Comparing Face Proportion Across Pre-sitters, Sitters, and Walkers in Middle and Top Areas of the Frame*

| Area | Comparison | *n*₁ | *n*₂ | Median₁ | Median₂ | *U* | *p* |
| --- | --- | --- | --- | --- | --- | --- | --- |
| Middle | Pre-sitters vs. Sitter | 4 | 8 | .08 | .02 | 25 | .154 |
|  | Pre-sitters vs. Walker | 4 | 17 | .08 | .05 | 53 | .099 |
|  | Sitter vs. Walker | 8 | 17 | .02 | .05 | 46 | .215 |
| Top | Pre-sitters vs. Sitter | 4 | 8 | .07 | .08 | 14 | .808 |
|  | Pre-sitters vs. Walker | 4 | 17 | .07 | .12 | 15 | .099 |
|  | Sitter vs. Walker | 8 | 17 | .08 | .12 | 42 | .140 |

*Note.* U = Mann–Whitney *U* statistic, two-tailed. Pre-sitters = unable to sit or walk; sitters = able to sit but unable to walk; walkers = able to sit and walk.
